# Pressure and Thermal Behavior of Elastic Polyurethane and Polyamide Knitted Fabrics for Compression Textiles

**DOI:** 10.3390/polym17070831

**Published:** 2025-03-21

**Authors:** Nga Wun Li, Mei-Ying Kwan, Kit-Lun Yick

**Affiliations:** 1 Faculty of Design, Architecture and Building, University of Technology Sydney, Ultimo, NSW 2007, Australia; 2 School of Fashion and Textiles, The Hong Kong Polytechnic University, Hung Hom, Kowloon, Hong Kong

**Keywords:** pressure distribution, thermal comfort, knitted pattern, seamless knitting, compression textiles

## Abstract

Compression stockings have long been manufactured in a single color without patterns, but enhancing their aesthetic appeal through knitted designs can improve user compliance. This study explores the potential of punch lace knitted structures to create patterns in compression textiles by seamless knitting technology while maintaining sufficient pressure. The effects of yarn material, number of yarns used, and knitted patterns on pressure and thermal comfort will be studied. The fabric pressure was evaluated using pressure sensors with a leg mannequin, while the thermal properties were measured according to the textile standard. This study found that the pressure and thermal conductivity of fabric are significantly influenced by the number of yarn and yarn materials, but not the knitted pattern. Cupro/cotton/polyurethane yarn (A) exhibits the strongest positive impact on pressure, increasing by 2.03 mmHg with the addition of one end of yarn A while polyamide/lycra yarn (C) exhibits a higher thermal conductivity than yarn A. For air permeability, the number of yarn and knitted patterns significantly affects the ventilation resistance. Pattern B with an additional needle in a float stitch shows 0.023 kPa·s/m lower resistance than pattern A. The findings from this study can be widely used in health, medical, and sports applications.

## 1. Introduction

Compression stockings have been proposed as a first line of treatment for a range of venous diseases as they are effective, safe, and inexpensive [1]. They exert maximum pressure on the ankles to achieve a leg pressure gradient and gradually decrease toward the knees to assist blood movement upwards towards the heart [2,3]. The applied pressure relies on the stockings’ fit. Circular and flatbed weft knitting technologies are widely used in the production of elastic compression textiles [4,5]. In circular knitting, the single jersey structure is commonly employed [6] to create lightweight, seamless compression stockings in plain colors without graphical patterns. In flatbed weft knitting, various structures—including single and double jersey, interlock, rib, and spacer fabrics—have been used in compression textiles, incorporating elastic yarns through inlay or plating techniques [5,7]. However, flatbed knitted fabrics require a sewing process, which can lead to an uneven pressure distribution due to seams, potentially compromising the effectiveness of compression therapy and wearer comfort. As a result, circular-knitted single-jersey compression stockings, designed with a single color, have dominated the market. Enhancing the aesthetic appeal of compression textiles through knitted graphical patterns remains largely unexplored, although it can improve user acceptance and compliance.

Over the past five years, weft-knitted seamless technology has advanced significantly, improving fashion sustainability by minimizing material waste and increasing production efficiency [8]. This technology enables rapid prototyping and made-to-measure production, which is particularly beneficial for compression garments, for which a precise fit is essential for optimal compression performance. Furthermore, recent advancements—such as the Shima Seiki SWG-XR seamless knitting machine (launched in 2022)—have expanded the capability of seamless knitting to incorporate complex structures and graphical patterns, including punch lace knitted structures. Unlike conventional double-knitted jacquard designs, which increase fabric thickness, punch lace structures allow for seamless graphic integration while preserving the lightweight nature of a single-jersey fabric. This breakthrough offers new opportunities to enhance both the functionality and aesthetics of compression textiles.

Previous studies have found that inappropriate materials and textile design can lead to improper pressure and excessive heat, resulting in discomfort, numbness to the leg, breathing difficulty, and even severe damage to one’s health [5,9]. The leggings knitted with yarn in a higher proportion of elastic polyurethane result in a higher level of wearer satisfaction due to the even pressure distribution and reduce the wearer’s sense of garment constraint [10]. A smaller loop length in the knitted fabric increases compression in plain and interlock fabrics [11] but reduces the compression in a partially plaited knit compared to jersey-plated fabric [12]. The rib structure in weft-knitted fabric provides the lowest thickness and pressure, while a full cardigan knitted structure exerts a higher level of compression than a half-cardigan knit structure [7]. In a fabric with elastane inlays, the increase in the yarn count of inlaid elastane is the most significant factor in the increasing pressure. The thicker the fabric, the higher the stitch density and fabric weight, and less traversal elasticity can create greater pressure [13]. However, the potential of punch lace knitted structures to provide pressure in compression textiles remains unexplored.

Apart from pressure performance, thermal properties like air permeability and thermal conductivity influence the comfort of compression stockings, while comfort is significantly affected by the fabric’s structural parameters [14,15]. Previous studies have found that knitted textiles’ thermal resistance is strongly correlated to the fabric thickness, mass per unit area, cover factor, and porosity but not to the fibre thermal conductivity [16]. Öner and Okur [17] found that the increase in the fabric tightness decreases its air permeability but increases its wicking ability. Although Stoffberg et al. [18] found that the fabric mass and thickness had a more significant effect on comfort-related properties than the fibre type and fabric structure, the increased spacer yarn density provides better air permeability [19]. Nevertheless, research on the pressure and thermal comfort of punch lace knitted structures incorporating polyurethane and polyamide yarns remains limited. Therefore, a deeper understanding of how the number of yarns used and knitting patterns influence pressure and thermal behavior is essential. This study systematically analyzes fabric structural parameters affecting compression performance, thermal conductivity, and air permeability. The findings provide valuable insights into the selection of compression textile materials, contributing to advancements in textile-based compression therapy.

## 2. Materials and Methods

### 2.1. Knitting Materials

A systematic investigation is conducted on the influence of yarn type, the number of yarns, and knitted patterns on compression pressure and thermal comfort properties. Three types of yarn were used, including (A) 2/93NM 55% cupro 45% cotton plied with one end of 30D degradable polyurethane (GSI Creos group, Tokyo, Japan), (B) 43D 69% nylon 31% Lycra (Daiya FUKUSHOKU Co., Ltd., Niigata, Japan), and (C) 94dtex 80% polyamide 6.6 20% Lycra (W. Zimmermann GmbH & Co. KG, Weiler-Simmerberg, Germany).

### 2.2. Fabrication of Knitted Samples

**Figure 1:**
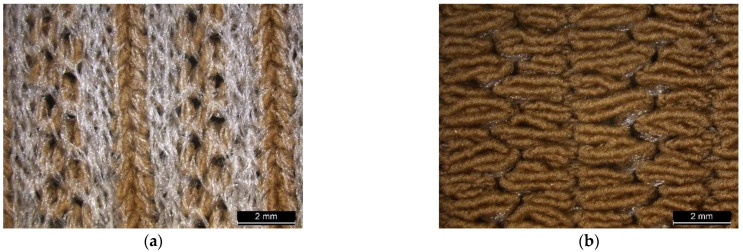
Microscopic view of fabric T6: (**a**) front and (**b**) back.

**Figure 2:**
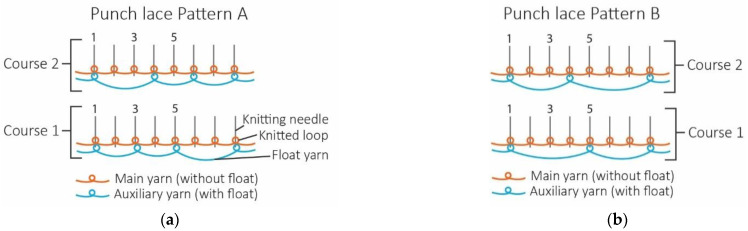
Knitting notations of fabrics with the punch lace knitted patterns (**a**) A and (**b**) B.

Six seamless knitted fabric samples with various yarn types, numbers of yarns, and knitted patterns were fabricated with a punch lace knitted structure, and one knitted fabric with a single-jersey structure was used as the control. All fabric samples were knitted on an 18-gauge seamless knitting machine (SWG-XR, SHIMA SEIKI MFG., Ltd., Wakayama, Japan). The punch lace structure was constructed by two different yarns knitting simultaneously, with the main yarn forming a knitted loop on all needles while the auxiliary yarn formed a knitted loop with selected needles and float stitch on the other needles. Two knitted patterns were selected. Pattern A consists of four needles with float stitches and four needles with knit stitches, while pattern B consists of five needles with float stitches and three needles with knit stitches. All samples were prepared using the same knitting tension and parameters and knitted in a tube form with 10 cm height and 6.5 cm width. Two specimens were made for each knitting condition. The details of the experiment’s design, fabric components, and sample specifications are provided in Table 1, Table 2 and Table 3. A microscopic view of punch lace knitted fabric is shown in Figure 1, while the knitting notations of the fabric with punch lace patterns A and B are illustrated in Figure 2.

### 2.3. Evaluation of Pressure and Thermal Behaviours of Fabric

Tests on the samples’ physical, pressure, and thermal comfort properties were conducted (Table 4) according to the textile standard. The fabric samples were conditioned for 24 h at a temperature of 20 ± 1 °C and relative humidity of 65% ± 5% before the testing occurred. Two samples were knitted with each type of fabric. Each sample was tested 3 to 6 times on different areas of the samples, and their mean value was calculated and used.

#### 2.3.1. Pressure

**Figure 3:**
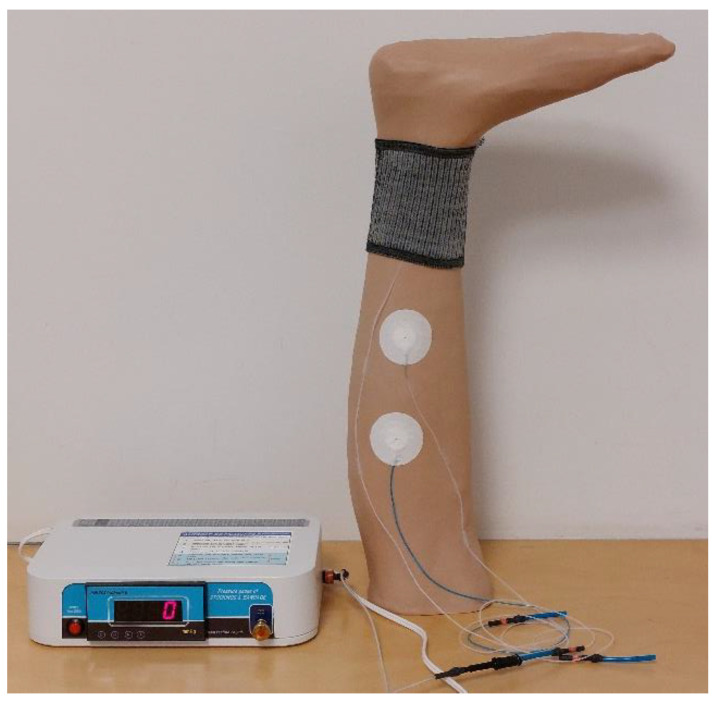
Evaluation of pressure exerted on the leg mannequin by knitted tube T5 with AMI air-pack pressure sensor on the ankle.

The pressure test was conducted on a 3D-printed leg mannequin with an ankle circumference of 21 cm and a calf circumference of 33.5 cm [24]. It was 3D printed using Big Rep One printer with polylactic acid (PLA) and covered with a 1 mm thick Pevalen™ prosthetic cover (Embreis AB, Stockholm, Sweden). The pressure of knitted tube samples was measured using an AMI air-pack pressure sensor (AMI3037-SB-SET, SANKO TSUSHO Co., Ltd., Tokyo, Japan) positioned on the ankle with a thin sensor bladder with thickness of 1 mm and diameter of 20 mm (Figure 3).

#### 2.3.2. Air Permeability

This test was conducted in a controlled laboratory environment maintained at 20 ± 1 °C and 65% ± 5% relative humidity. The air permeability is evaluated using the KES-F8-AP1 air permeability tester (KATO TECH Co., Ltd., Kyoto, Japan), which measures ventilation resistance. Fabric with smaller ventilation resistance values means higher levels of breathability and permeability.

#### 2.3.3. Thermal Conductivity

**Figure 4:**
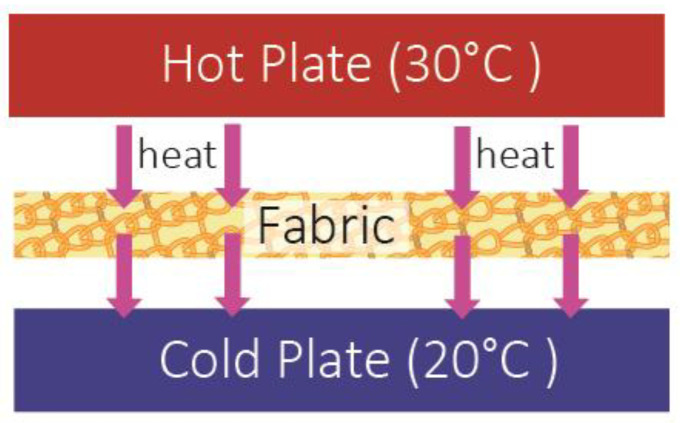
The thermal network diagram of the thermal conductivity test using hot plates.

The thermal conductivity test was conducted in a controlled laboratory environment maintained at 20 ± 1 °C and 65% ± 5% relative humidity. A 10 cm × 10 cm fabric sample was placed between two heat plates set at constant temperatures of 30 °C and 20 °C, following the JIS L 1927 standard. The heat transmitted through the sample due to the temperature difference was measured (Figure 4). Each fabric sample was tested in 60 s. The thermal conductivity is calculated by using Equation (1):

k = (W × D)/(A × ∆T)(1)

where k is the thermal conductivity (W/cm °C), W is the heat transmitted through the sample (W), D is the thickness of the sample (cm), A is the area of the heat plate (25 cm^2^), and ∆T is the temperature difference (10 °C).

### 2.4. Statistical Analysis

The experimental data were analyzed by using R programming. Kruskal–Wallis test and Dunn’s test were conducted to test the significant differences. Multiple linear regression was adopted to investigate the relationship between the four different independent variables: (1) Number of yarn A, (2) Number of yarn B, (3) Number of yarn C, and (4) knitted pattern and three dependent variables (pressure, air permeability, and thermal conductivity). Before the analysis, the values were checked by the normal Q-Q plots and Shapiro–Wilk tests. The significance level of the statistical analysis was set at a level of 0.05.

## 3. Results and Discussion

**Figure 5:**
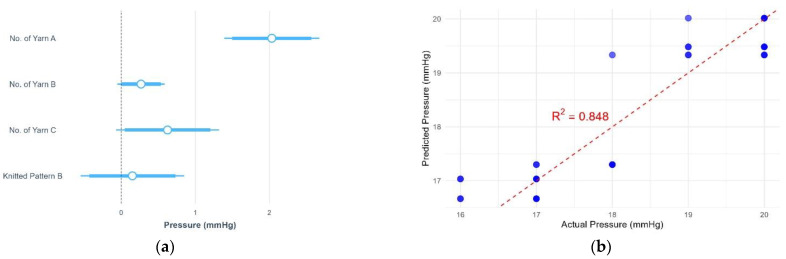
(**a**) Statistical summary of pressure; (**b**) scatter plot of actual and predicted pressure.

**Figure 6:**
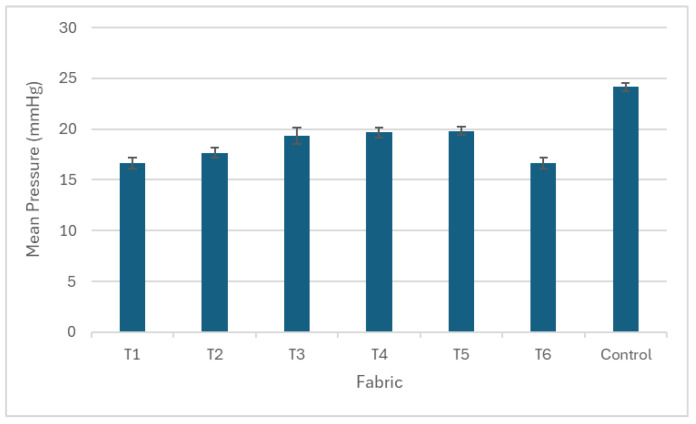
The pressure of the punch lace fabrics (T1–T6) and the control fabric.

**Figure 7:**
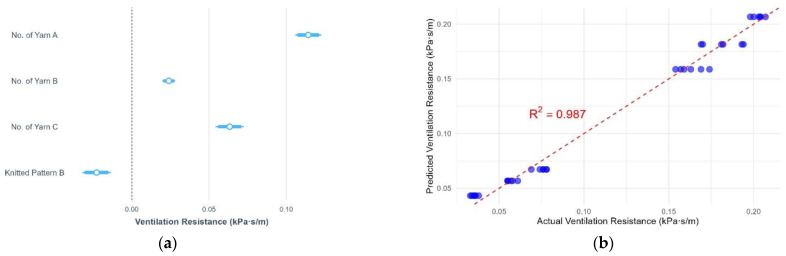
(**a**) Statistical summary of ventilation resistance; (**b**) Scatter plot of actual and predicted ventilation resistance.

**Figure 8:**
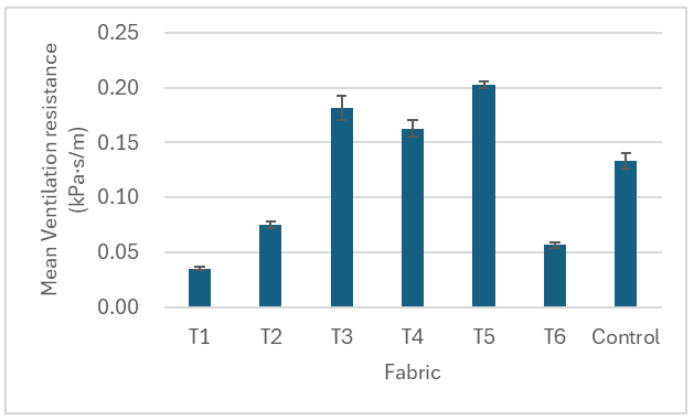
Air permeability of the punch lace fabrics (T1–T6) and the control fabric.

**Figure 9:**
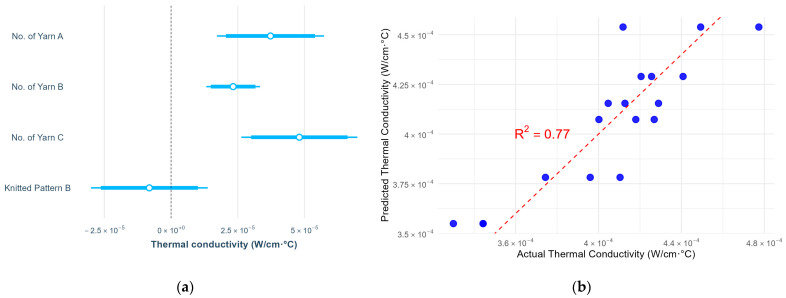
(**a**) Statistical summary of thermal conductivity; (**b**) Scatter plot of actual and predicted thermal conductivity.

**Figure 10:**
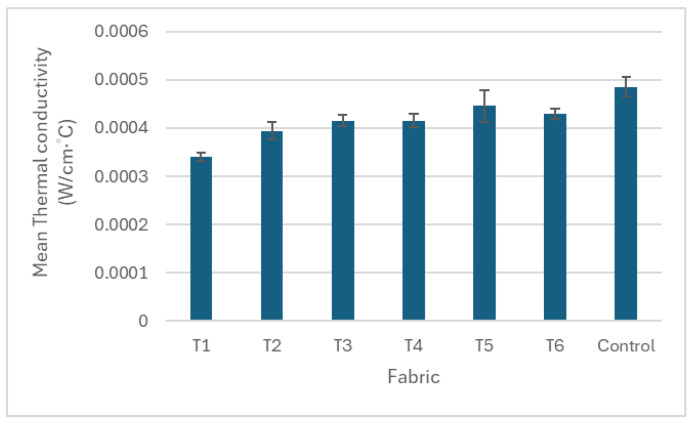
Thermal conductivity of the punch lace fabrics (T1–T6) and the control fabric.

This study examined the effects of yarn material, number of yarns used, and knitted patterns on pressure, thermal comfort and air permeability. The results of pressure, air permeability and thermal comfort are illustrated in Figure 5, Figure 6, Figure 7, Figure 8, Figure 9 and Figure 10. A summary of research findings with the comparison of previous research is provided in Table 5.

### 3.1. Pressure

The result of the multiple regression analysis revealed that the changes in the number of cupro/cotton/polyurethane yarns (A), nylon/Lycra yarns (B), and polyamide/Lycra yarns (C) significantly impacted the pressure (*p* < 0.05) but not the knitted pattern. The pressure value of the fabric is significantly affected by the number of yarn A, followed by yarn C and yarn B. The results of the multiple linear regression found that the pressure increased by 2.03 mmHg when adding one end of yarn A. When adding one end of yarn C, the pressure rises by 0.63 mmHg, while the pressure only increases by 0.27 mmHg with one more yarn B (Figure 5a). Although the knitted pattern has an insignificant effect on the pressure, the pressure increases by 0.15 mmHg in pattern B when compared with the fabric in pattern A (*p* > 0.05). These results indicate that the higher pressure is a function of more yarn A, B, and C in the fabric. Overall, the model explains 84.77% of the variance in pressure (*p* < 0.05). The regression equation for the fabric’s pressure is as follows:

Fabric’s pressure in pattern B = 14.88 + 2.03 × N_A_ + 0.27 × N_B_ + 0.63 × N_C_ (2)

where N_A_ is the number of yarn A, N_B_ is the number of yarn B, and N_C_ is the number of yarn C. The result predicted by the regression equation is valid, as a comparison of the predicted and actual pressure yields R^2^ = 0.85 (Figure 5b).

Among the punch lace fabrics shown in Figure 6, T5 has the highest pressure with 19.83 mmHg at the ankle point, followed by T4 (19.67 mmHg) and T3 (19.33 mmHg), while T6 has the lowest pressure (16.67 mmHg). This can be explained by the fact that T3 and T4 contain two ends of cupro/cotton/polyurethane yarn (A) and two ends of nylon/Lycra (B), while T5 includes two additional ends of nylon/Lycra yarn (B), resulting in a total of four ends of nylon/Lycra (B). This gives T5 the highest number of yarns among the six punch lace knitted fabrics. The additional nylon/Lycra (B) slightly increases the fabric pressure; however, the difference between T3 and T4 is minimal, at approximately 0.34 mmHg, while the difference between T4 and T5 is 0.16 mmHg. Comparing T4 and T6, both have four yarn ends, yet T6 exhibits the lowest pressure (16.67 mmHg), while T4 reaches 19.67 mmHg. The 3 mmHg pressure difference suggests that replacing two ends of elastic polyamide/Lycra (C) with two ends of cupro/cotton/polyurethane (A) increases the pressure by 18%. This indicates that yarn material plays a more significant role in pressure variation than the number of yarns in the fabric.

When looking into the yarn material, yarn A is made of one end of cupro and cotton blend yarn ply with one end of 30D degradable polyurethane, while the polyamide/Lycra yarn (C) is a 94dtex (equal to 85.5D) elastane made of polyamide and Lycra with 100% elongation. A higher denier number in polyamide/Lycra yarn (C) indicates a thicker yarn. Polyurethane is the only elastane in yarn A, and it is approximately three times finer than yarn C. It can be predicted that yarn C has a higher elasticity than yarn A, which increases the elasticity of fabric T6 and provides less pressure. This result is consistent with a previous study [13] that stated that the traversal elasticity has the strongest negative effect on fabric pressure.

Moreover, T3 and T4 have a similar pressure, although T3 in knitted pattern A has four needles in the knitting float stitch per repeated pattern while T4 has five needles. Different from the previous study [25], these results show that the float length in the punch lace knitted structure has insignificant effects on fabric pressure.

Interestingly, the control fabric exhibits the highest pressure among all knitted samples (T1–T6). It is knitted in a single-jersey structure with one end of cupro/cotton/polyurethane yarn (A) and one end of nylon/Lycra yarn (B), in which all needles form loops without float stitches. The elastic yarn knitted in float stitches in the punch lace knitted structure enhances fabric elasticity. In contrast, the single-jersey structure, with more knitted loops, restricts elasticity, leading to a higher pressure. Comparing T1 (the punch lace structure) and the control fabric (the single-jersey structure), the control fabric has a higher weight but a similar thickness to T1, despite being knitted with the same yarn type and number of yarns. This is due to the greater number of knitted loops, which increases yarn usage and, consequently, fabric weight. This explains why compression stockings are commonly made with a single-jersey structure, as seen in the control fabric, to provide a higher degree of pressure to the body with a similar fabric thickness. However, this structure is stiffer and less elastic, making it more difficult to put on and off compared to punch lace knitted fabrics.

### 3.2. Air Permeability

When investigating air permeability, the smaller values of ventilation resistance mean higher levels of breathability and permeability. This study found that over 98% of the variance in ventilation resistance is accounted for by the number of yarns and the knitted pattern (*p* = 2.2 × 10^−16^). Like the fabric pressure, the number of yarn A shows the strongest positive effect on the ventilation resistance, followed by yarn C and B. The ventilation resistance increased by 0.11 kPa·s/m, 0.06 kPa·s/m, and 0.02 kPa·s/m when the fabric added one end of yarn A, C, and B, respectively (Figure 7a). It indicated that the increase in the number of yarns lowered the air permeability of the fabric. When the fabric is knitted in pattern B, the ventilation resistance decreases by 0.023 kPa·s/m compared with pattern A. The fabric knitted in pattern B is more breathable than in pattern A. The comparison of the predicted and actual ventilation resistance yields R^2^ = 0.99 (Figure 7b). The regression equation for the fabric’s air permeability is as follows:

Fabric’s air permeability in pattern A = −0.095 + 0.11× N_A_ +0.02 × N_B_ + 0.06 × N_C_ (3)

Fabric’s air permeability in pattern B = −0.072 + 0.11× N_A_ +0.02 × N_B_ + 0.06 × N_C_ (4)

where N_A_ is the number of yarn A, N_B_ is the number of yarn B, and N_C_ is the number of yarn C.

The ventilation resistance increases by adding the number of yarns A and B in fabrics T1 to T3 (Figure 8). The air is obstructed from passing through the fabric when the yarn number increases. This is due to the increased fabric thickness when adding extra yarn while knitting in the same loop length. The ventilation resistance of T4 knitted with pattern B is lower than T3 in pattern A, which is due to the additional one needle with the float stitch in pattern B. A longer float length in the knitted structure facilitates greater airflow through the fabric, thereby enhancing its air permeability. When comparing T4 and T6, the knitted yarn changed from yarn A to yarn C, resulting in a significant drop in ventilation resistance in T6. This can be explained by the fact that fabric T6 is 43% less thick and 35% lighter when compared with T4 (Table 3). This indicates that the fabric with a lower thickness and weight resulted in a smaller ventilation resistance, as the air can easily pass through the fabric with less obstruction by the yarn, thus increasing air permeability [15].

Among the six punch lace knitted fabrics, T1 has the lowest and T5 the highest ventilation resistance. T1 is knitted with one end of cupro/cotton/polyurethane yarn (A) and one end of nylon/Lycra yarn (B) in pattern A. It has the lowest number of yarns, resulting in the lightest fabric weight and a loose structure compared to other samples. Despite having one less needle in the float stitch and ranking third in fabric thickness, T1 exhibits the lowest ventilation resistance, indicating the highest air permeability. In contrast, T5 is knitted with six ends of yarn, leading to the heaviest fabric weight and greatest thickness. Although T5 is knitted in pattern B, which has one additional needle in the float stitch compared to pattern A, it still exhibits the lowest air permeability. Previous studies have stated that air permeability is closely related to the pattern of the knitted fabric and the insertion density of the inlay yarn [26]. This result further suggests that air permeability is primarily influenced by the number of yarns and fabric weight, followed by the fabric thickness, with the knitted pattern having less impact.

### 3.3. Thermal Conductivity

Unlike the air permeability, the number of polyamide/Lycra yarns (C) shows the strongest positive effect on thermal conductivity, followed by the cupro/cotton/polyurethane yarn (A) and nylon/Lycra yarn (B). Different from the previous study [27], a similar thermal conductivity is found in knitted patterns A and B (Figure 9). This study found that 77% of the variance in thermal conductivity is accounted for by the number of yarns A, B, and C, but no significant difference is found with the knitted pattern (*p* > 0.05). The increase in the number of yarns significantly increased the thermal conductivity of the fabric. The regression equation for the fabric’s thermal conductivity is as follows:

Fabric’s thermal conductivity in pattern B = 3.02534 × 10^−4^ + 3.726 × 10^−5^ × N_A_ + 2.327 × 10^−5^ × N_B_ + 4.808 × 10^−5^ × N_C_ (5)

where N_A_ is the number of yarn A, N_B_ is the number of yarn B, and N_C_ is the number of yarn C.

The thermal conductivity gradually increases with the number of yarns A and B from T1 to T5 (Figure 10), where T1 is constructed with two yarn ends and T5 with six ends. Fabrics T3 and T4 exhibit a similar thermal conductivity, as both contain four yarn ends, despite differences in knitted patterns and float lengths. This trend is attributed to the increased surface area from additional yarns, enhancing heat transfer through conduction. Meanwhile, knitted pattern variations have a minimal impact on thermal conductivity. Similar to the previous study [19], the thermal conductivity of punch lace knitted fabrics can be affected by yarn type. Comparing yarn types, T6 (knitted with two ends of nylon/Lycra (B) and two ends of polyamide/Lycra (C)) exhibits a higher level of thermal conductivity than T4 (knitted with two ends of nylon/Lycra (B) and two ends of cupro/cotton/polyurethane (A)), both using pattern B. This indicates that polyamide/Lycra (C) conducts heat more effectively than cupro/cotton/polyurethane (A). The findings confirm that yarn material and the number of yarns used in the fabric are key factors influencing fabric thermal conductivity.

Interestingly, the control fabrics knitted with a single-jersey structure had the highest thermal conductivity. When comparing fabric T1 and the control, both have the same number of yarns and materials but differ in knitted structures. In T1, cupro/cotton/polyurethane (A) forms float stitches in the punch lace knitted structure, whereas in the control fabric, all the needles create knitted loops in a single-jersey structure. The higher fabric weight of the control fabric than that in fabric T1 confirms that the float stitches in T1 require less yarn compared to the knitted loops in the control fabric. With less yarn in contact with heat, T1 transfers less heat through conduction, resulting in a lower thermal conductivity.

Among the punch lace fabrics, fabric T6 has the second highest thermal conductivity and excellent air permeability performance, but the least pressure. When looking at T5, it can offer the highest pressure on the leg mannequin and the best performance in thermal conductivity. However, it has the lowest air permeability. This study found that balancing the need to acquire a higher pressure while providing more thermal comfort in compression textiles is challenging. Meanwhile, compression garments are made of textiles with a certain degree of stretching to offer compression pressure to the body. This stretchy feature changes the porosity of fabrics, which influences the air permeability and the wicking properties of the garment [28]. Further studies should be conducted to optimize the compression function while balancing thermal comfort in compression textiles in seamless knitting.

**Table 5:** Summary of research findings with comparison of previous research.

Tested Properties	Consistent with or Different from Previous Studies	Reference Study	Key Findings in this Study
Pressure	Consistent	Sarı & Oğlakcıoğlu (2018) [13]	Polyamide/lycra yarn (C) has higher elasticity than cupro/cotton/polyurethane yarn (A), which increases the elasticity of fabric T6 and provides less pressure in fabric T6.
	Different	Maqsood et al. (2016) [25]	The float length in the punch lace knitted structure has insignificant effects on fabric pressure.
Air permeability	Consistent	Kaplan & Akgünoğlu (2021) [15]	The fabric with lower thickness and weight resulted in a smaller ventilation resistance, as the air can easily pass through the fabric with less obstruction by the yarn, thus increasing air permeability.
	Different	Muraliene & Mikucioniene (2020) [26]	Air permeability is primarily influenced by the number of yarns and fabric weight, followed by fabric thickness, with the knitted pattern having less impact.
Thermal conductivity	Consistent	Li et al. (2022) [19]	The thermal conductivity of punch lace knitted fabrics can be affected by yarn type.
	Different	Kwan et al. (2024) [27]	No significant difference is found in thermal conductivity with knitted patterns A and B (with extra one float stitch)

## 4. Conclusions

This study proposes the use of knitted punch lace structures for compression textiles and systematically investigates the effects of yarn material, the number of yarns, and knitted patterns on the fabric’s pressure and thermal behavior. The following conclusions are drawn based on the experimental results.

- The pressure of the fabric is significantly influenced by the number of yarns and yarn materials, but not the knitted pattern. Among the three yarns, cupro/cotton/polyurethane yarn (A) exhibits the strongest positive impact on pressure, increasing by 2.03 mmHg with the addition of one end of yarn A. This is attributed to the fine 30D Degradable polyurethane in yarn A, which is the only elastane component in this yarn, reducing the fabric elasticity. The results indicate that a lower fabric elasticity leads to a higher fabric pressure;
- For air permeability, the number of yarns and knitted patterns significantly affect the ventilation resistance. Cupro/cotton/polyurethane yarn (A) exhibits the strongest effect, increasing ventilation resistance by 0.11 kPa·s/m per added end, thereby reducing air permeability. In contrast, pattern B shows a 0.023 kPa·s/m lower resistance than pattern A, as its additional needle with a float stitch facilitates greater airflow. The air permeability is primarily influenced by the number of yarns and fabric weight, followed by the fabric thickness and the knitted pattern. Therefore, the fabric with a lower weight and thickness has higher levels of air permeability as the air can easily pass through the fabric with less obstruction by the yarn;
- For thermal conductivity, an increase in yarns A, B, and C has a significant positive effect, while knitted patterns have no noticeable impact. This is attributed to the larger surface area created by additional yarns, which enhances heat transfer through conduction via the yarn material. Among the yarn types, polyamide/lycra (C) exhibits a higher thermal conductivity than cupro/cotton/polyurethane (A). The findings confirm that the yarn material and the number of yarns used in the fabric are key factors influencing the thermal conductivity of the fabric;
- Among the six punch lace fabrics, fabric T6 knitted with polyamide/lycra yarn (C) has good thermal conductivity and excellent air permeability but the lowest pressure. T5 knitted with cupro/cotton/polyurethane yarn (A) can deliver the highest pressure and good thermal conductivity but poor air permeability. This study found that finding a balance between acquiring a higher pressure and providing more thermal comfort in compression textiles is challenging.

A thorough understanding of the structural parameters that affect pressure and thermal comfort performance is crucial for advancing the design of compression textiles. Optimizing these parameters can enhance both pressure effectiveness and wear comfort, which holds significant clinical relevance for medical compression garments used in managing conditions such as venous disorders, lymphedema and post-surgical recovery. Additionally, these insights can inform the development of athletic wear for muscle support and performance enhancement as well as therapeutic textiles for rehabilitation and injury prevention. By refining yarn and fabric structures, compression textiles can be better tailored to diverse functional and medical needs.

## Figures and Tables

**Table 1:** Design of experiment.

Factor	Level		
Yarn material	(A) 2/93NM 55% cupro 45% cotton + 30D degradable polyurethane	(B) 43D 69% nylon 31% Lycra	(C) 94dtex 80% polyamide 6.6 20% Lycra
Number of yarns	1	2	4
Knitted pattern	A	B	/

**Table 2:** Fabric component.

Fabric Component	T1–T4	T5	T6
Main yarn (without float)	Yarn B	Yarn B (2 Ends)	Yarn B
Auxiliary yarn (with float)	Yarn A	Yarn A (2 Ends) + Yarn B (2 Ends)	Yarn C

**Table 3:** Sample specifications of punch lace knitted specimens (T1–T6) and control fabric.

Fabric Code	Number of Yarns			Knitted Pattern	Fabric Weight (g/m^2^)	Thickness (mm)
	Yarn A	Yarn B	Yarn C			
T1	1	1	/	A	249.80	1.79
T2	1	2	/	A	281.63	1.80
T3	2	2	/	A	405.71	2.02
T4	2	2	/	B	406.53	2.22
T5	2	4	/	B	463.67	2.34
T6	/	2	2	B	266.12	1.27
Control	1	1	/	/	292.24	1.60

**Table 4:** Summary of test methods.

Property	Device	Testing Standard
Thickness	Thickness gauge (Model BC1110-1-04, AMES LOGIC Basic, Chicopee, MA, USA)	ASTM D1777 standard test method for thickness of textile materials [20]
Pressure	Leg mannequin with an AMI air-pack pressure sensor (AMI3037-SB-SET, SANKO TSUSHO CO., LTD, Tokyo, Japan)	CEN/TF 15,831 method for testing compression in medical hosiery [21]
Air permeability	Air permeability tester (KES-F8-AP1, KATO Tech Co., Ltd., Kyoto, Japan)	ASTM-D737-18 standard test method for air permeability of textile fabrics [22]
Thermal conductivity	Thermal measuring unit (KES-F7 Thermo Labo II, KATO Tech Co., Ltd., Kyoto, Japan)	JIS L 1927 standard test for textile measurement method of cool touch feeling property [23]

## Data Availability

Data are contained within the article.
